# Cytophilic antibodies to *Plasmodium falciparum *Glutamate Rich Protein are associated with malaria protection in an area of holoendemic transmission

**DOI:** 10.1186/1475-2875-4-48

**Published:** 2005-09-29

**Authors:** John PA Lusingu, Lasse S Vestergaard, Michael Alifrangis, Bruno P Mmbando, Michael Theisen, Andrew Y Kitua, Martha M Lemnge, Thor G Theander

**Affiliations:** 1 National Institute for Medical Research, Amani Medical Research Centre, Tanga & Headquarters, Dar es Salaam, Tanzania; 2 Centre for Medical Parasitology, Institute of Medical Microbiology and Immunology, University of Copenhagen and Department of Infectious Diseases, Copenhagen University Hospital (Rigshospitalet), Denmark; 3 Statens Serum Institut, Copenhagen, Denmark

## Abstract

**Background:**

Several studies conducted in areas of medium or low malaria transmission intensity have found associations between malaria immunity and plasma antibody levels to glutamate rich protein (GLURP). This study was conducted to analyse if a similar relationship could be documented in an area of intense malaria transmission.

**Methods:**

A six month longitudinal study was conducted in an area of holoendemic malaria transmission in north-eastern Tanzania, where the incidence of febrile malaria decreased sharply by the age of three years, and anaemia constituted a significant part of the malaria disease burden. Plasma antibodies to glutamate rich protein (GLURP) were analysed and related with protection against malaria morbidity in models correcting for the effect of age.

**Results:**

The risk of febrile malaria episodes was reduced significantly in children with measurable anti-GLURP IgG1 antibodies at enrolment [adjusted odds ratio: 0.39 (95% CI: 0.15, 0.99); *P *= 0.047]. Interestingly, there was an inverse relationship between the plasma anti-GLURP IgG1 and IgG3 levels and the levels of parasitaemia at enrolment. However, anti-GLURP IgG2 and IgG4 levels were not associated with reduction in parasite density. Similarly, antibody levels were not associated with haemoglobin levels or anaemia risk.

**Conclusion:**

Cytophilic IgG1 and IgG3 antibodies against R0-GLURP may contribute to the control of parasite multiplication and reduction in febrile malaria incidence in children living in an area of intense malaria transmission.

## Background

In areas of stable malaria transmission, immunity is acquired during childhood [1,2], and the protection is mainly mediated by antibodies directed against the blood stages of the parasite [3]. The relationship between malaria morbidity and antibody levels to malaria antigens has been analysed in several prospective longitudinal studies performed in different parts of Africa and Asia [4-9]. The Glutamate Rich Protein (GLURP) is a *Plasmodium falciparum *antigen, which has been studied extensively. It is a 220 kD protein expressed in the hepatic, asexual and sexual stages of the parasite life cycle [10]. The protein can be divided into an N-terminal non-repeat region (R_25–500 _or R0), a central repeat region (R1) and a C-terminal repeat region (R2) [11]. GLURP is a malaria vaccine candidate, which has undergone phase 1 trials in Europe and trials are planned to take place in Africa in the near future.

Several immuno-epidemiological studies using sera and clinical data from various sites have consistently identified high anti-R0-GLURP immunoglobulin G (IgG) levels as significant predictors of protection against high levels of parasitaemia, and febrile malaria episodes [6,12-16]. The protective antibodies are thought to elicit antibody dependent cytotoxic inhibition (ADCI) [17] through binding to the surfaces of merozoites [18]. Most of these studies have been performed in areas of moderate malaria transmission where protection against malaria fevers is achieved in those aged 5–15 years. In this report, plasma antibody levels to R0-GLURP was measured and related to malaria morbidity in a village subjected to holoendemic transmission and entomological inoculation rates exceeding one infectious bite per night [2]. In this community the incidence of febrile malaria decreases sharply by the age of three years and anaemia constitutes a significant part of the malaria disease burden [19]. Antibody levels to R0-GLURP in two other villages located in areas of moderate and low transmission were measured to compare the age related acquisition of antibodies in individuals living under different malaria transmission intensity.

## Materials and methods

### Study sites and population

A longitudinal malariometric study was carried out in three villages with different malaria transmission intensity in the Tanga region, Tanzania, as described in detail elsewhere [19]. The villages are situated at varying altitudes, which in north-eastern Tanzania is a proxy for malaria transmission intensity [20]. Malariometric surveys were conducted and blood samples were collected in April, July and September. Haemoglobin levels were measured using a HemoCue^® ^photometer (Ångelholm, Sweden) and thick and thin blood smears for malarial microscopy were prepared. Thereafter, blood was centrifuged to obtain plasma, which was frozen at -20°C. Local village helpers and health workers at nearby health facilities performed passive case detection during the six month study period. The village helpers were provided with first-line antimalarial drug (sulphadoxine-pyrimethamine), paracetamol, microscope slides, blood lancets, treatment charts, febrile case detection forms and storage boxes. Villagers could seek treatment at any time from these helpers. Patients with symptoms of malaria were treated with the first-line antimalarial drug. If they had severe symptoms or did not respond adequately to the first-line treatment, they were referred to a health facility. Prior to treatment, the village helpers collected clinical information and a malaria blood smear. At each nearby health facility, two permanent staff members monitored study participants seeking medical treatment at the facility. If study participants presented at the facility with a history of fever, a form was completed and a blood smear collected. Active febrile case detection was undertaken once per month by the research team. During active case detection, study participants were seen by a trained physician and a blood smear was taken from all study participants who had reported a history of fever within two days and/or had axillary temperature ≥ 37.5°C.

### Case definitions and selection of plasma samples for antibody assays

Febrile malaria episodes were defined as an axillary temperature ≥ 37.5°C and/or a history of fever within the previous 48 hours in the presence of asexual *P. falciparum *parasites ≥ 5000 parasites/μl [19]. Anaemia was defined as haemoglobin < 11.0 g/dl [21].

The incidence of febrile malaria episodes was low in Magamba and Ubiri [19]. In Mgome, 219 of the 254 individuals completed the longitudinal follow-up and from 171 of these individuals, sufficient plasma was available to measure R0-GLURP IgG class and subclass levels. Thus, antibody levels were measured in 9, 24, 33, 52, 35, and 18 individuals belonging to the 0–11 months, 1–2 years, 3–4 years, 5–9 years, 10–14 years, and 15–19 years age groups, respectively. Of the 171 individuals, 54 had febrile malaria episodes and 44 developed anaemia during the follow-up period.

To compare age-specific acquisition of R0-GLURP IgG class antibodies in areas of different endemicity, plasma samples of 40 individuals from Ubiri village (moderate transmission) and Magamba village (low transmission) were also tested. The samples were selected randomly from asymptomatic individuals to represent four age groups (0–4, 5–9, 10–14 and 15–19 years, N = 10 in each group). Since malaria morbidity was low in Ubiri and Magamba, no attempt was made to relate morbidity and anti-R0-GLURP IgG levels in these villages.

### Antibody assays

Antibodies to R0-GLURP were measured by enzyme-linked immunosorbent assay (ELISA) based on a protocol developed by Afro Immunoassay. Briefly, microtitre plates (Maxisorp Nunc, Roskilde, Denmark) were coated overnight at 4°C with purified his-tag produced recombinant R0-GLURP (0.5 μg/ml) diluted in phosphate buffered saline (PBS). The plates were blocked with 3% powdered-milk-containing-phosphate buffer for one hour. Plasma from samples diluted 1:200 in dilution buffer (PBS with 1% powdered-milk and 0.1% Tween-20) was added in duplicate. The plates were then incubated at room temperature for one hour, where after peroxidase-conjugated rabbit anti-human IgG or IgM (Dako, Glostrup, Denmark) was added. Plates were washed four times with washing buffer (PBS with 0.1% Tween-20 and 0.5 M NaCl) between steps. Colour was developed using hydrogen peroxide with *O*-phenylenediamine (Dako, Glostrup, Denmark), and reading of antibody absorbance was done at 492 nm. Samples were retested if the measured differences in absorbance values between duplicate samples were higher than 15%. For determination of IgG subclasses (IgG1, IgG2, IgG3 and IgG4), plasma samples diluted 1:50 were added in duplicate and incubated for one hour at room temperature. The following monoclonal mouse anti-human subclasses were used: clone NL16 for IgG1 (Sky lab), clone NP6002 for IgG2 (Sigma), clone ZG4 for IgG3 (Sky lab) and clone RJ4 for IgG4 (Sky lab). The monoclonal IgG subclasses were diluted 1:2000 for IgG1, 1:3000 for IgG2, IgG3 and IgG4 in dilution buffer and incubated for one hour at room temperature. Goat anti-mouse IgG conjugated to peroxidase (Caltag) diluted 1:3000 in dilution buffer was then added and plates incubated for one hour. Colour development and reading of antibody absorbance was done as described above. Antibody levels were measured relative to the titration of IgG and IgG1-4 standard solutions. Plasma samples from 31 adult healthy Danes without any previous exposure to malaria were used as negative controls to generate cut-off values, which was defined as mean plus two standard deviations for the respective IgG class or subclasses. A positive control containing a pool of plasma from adult Liberian individuals (kindly provided by Dr. S. Jepsen, Statens Serum Institut, Copenhagen, Denmark through Afro Immuno Assay) were tested in parallel to the study samples. The positive plasma pool contained 27.7, 48.7, 35.7, 18.5, and 46.6 arbitrary units (AU)/ml anti-R0GLURP antibodies of IgG, IgG1, IgG2, IgG3, and IgG4, respectively. The cut-off values in arbitrary units were 3.5, 1.4, 1.4, 1.7, and 0 AU/ml for IgG, IgG1, IgG2, IgG3, and IgG4, respectively. The highest OD values in positive samples were 2.7 for IgG and 3.8 for IgG1-4.

### Data analysis

Data were analysed using Stata/SE version 8.2 (Stata Corporation, Texas, USA; ). The age dependence of antibody responses was analyzed by use of Spearman's rank-order correlation and the mean matched-pairs test was used to compare R0-GLURP IgG levels between samples collected at the initiation and at the end of the study. The Mann-Whitney test was used to test R0-GLURP IgG class and subclasses levels between females and males. Univariate and multivariate models were fitted to estimate to what extent R0-GLURP IgG class and subclass levels could be attributed to protection against high parasite density, febrile malaria episodes or anaemia in individuals from the high transmission village. Differences were considered statistically significant if the 95% confidence interval was not overlapping or *P *< 0.05.

## Results

### Plasma antibody levels to R0-GLURP in areas of different malaria endemicity

**Figure 1:**
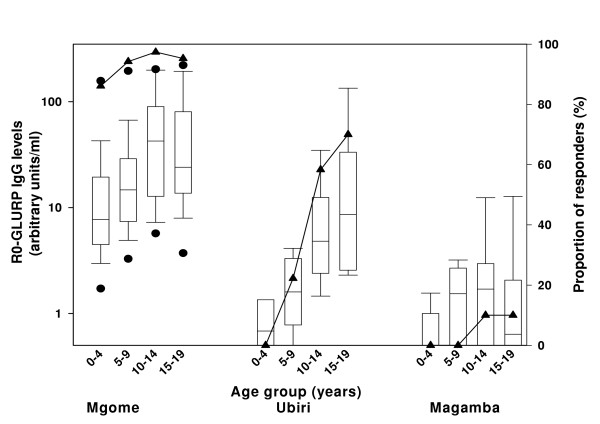
Age-specific IgG responses to recombinant R0-GLURP in three villages: Mgome (high malaria transmission), Ubiri (moderate malaria transmission) and Magamba (low malaria transmission). The levels are presented as arbitrary units on a logarithmic scale. Box plots illustrate medians with 25^th^, 75^th ^and whiskers for 10^th ^and 90^th ^percentiles including outliers (5^th^/95^th ^percentiles). Lines with filled triangles represent proportion of responders in percentages.

The prevalence and mean levels of anti-R0-GLURP IgG were highest in residents living under intense and holoendemic malaria transmission in Mgome, lower in individuals living under moderate and seasonal transmission in Ubiri and lowest in residents living under low and unstable transmission in Magamba (Figure 1). The differences in IgG levels between these villages were highly significant (P(z) < 0.001, Trend test). The prevalence and levels of antibodies increased with donor age in Mgome and Ubiri villages (Figure 1), although there was a tendency of slight decrease in antibody levels after the age of 14 years in Mgome. Antibody levels did not differ significantly between males and females (Mann-Whitney test Mgome P(z) = 0.40; Ubiri P(z) = 0.12.; Magamba P(z) = 0.94). In Mgome, there was no statistical difference in antibody levels between samples collected at the beginning and at the end of the study (mean difference [95% confidence interval (CI)]: 4.0 arbitrary units (AU) [-15.8, 23.9], P(t) = 0.68, paired T-test). The mean antibody levels were slightly higher in samples collected at the end of the study in Ubiri (mean difference [95%CI]: 6.4 AU [-2.1, 15.0], P(t) = 0.13, paired T-test) and Magamba (mean difference [95%CI]: 7.6 AU [6.2, 9.0], P(t) < 0.0001, paired T-test).

In all three villages, R0-GLURP IgM was detected at very low levels with no distinct patterns observed with respect to age, sex or season (data not shown).

### IgG subclass responses to R0-GLURP in Mgome village

**Figure 2:**
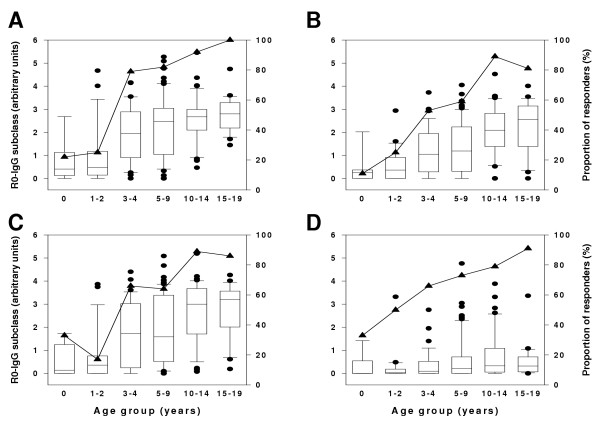
Age-specific IgG subclass responses to recombinant R0-GLURP in Mgome (high transmission village). Panels A, B, C and D represent IgG1, IgG2, IgG3, and IgG4 subclasses, respectively. The levels are presented as arbitrary units on a logarithmic scale. Box plots illustrate medians with 25^th^, 75^th ^and whiskers for 10^th ^and 90^th ^percentiles including outliers for each IgG subclass. Lines with filled triangles represent prevalence of responders in percentages.

The prevalence and mean levels of R0-GLURP IgG antibodies increased with age for all subclasses. For the cytophilic IgG1 and IgG3, there was a marked increase in three to four year old children (Figure 2). For the non-cytophilic IgG2 and IgG4, there was a steady increase in the levels and proportion of responders throughout the age groups (Figure 2). The levels of IgG4 antibodies were generally low.

### Relationship between R0-GLURP IgG levels and *Plasmodium falciparum *density in Mgome (high transmission village)

Multiple linear regression models adjusting for age were generated to determine whether the anti-R0-GLURP IgG levels were associated with *P. falciparum *density at the initiation of the study. Interestingly, there was a significant association between cytophilic IgG subclass antibodies and reduced parasite density. For IgG1, one log unit increase in antibodies was associated with a reduction in *P. falciparum *density by 1,401 parasites/μl [95% CI: 231, 2571; *P *= 0.019]; and for IgG3 level, one log unit increase was associated with a reduction in *P. falciparum *density at enrolment by 983 parasites/μl [95% CI: 126, 1841; *P *= 0.025]. Although similar associations were observed in univariate analysis for non-cytophilic IgG subclasses such associations were not significant after adjusting for age (Table 1).

**Table 1:** Relationship between *P. falciparum *density (parasites/μl) and anti R0-GLURP IgG levels (log arbitrary unit/ml) at enrolment.

R0-GLURP antibodies	Unadjusted coefficients (95% CI)^1^	*P*-value	Adjusted coefficients (95% CI)^2^	*P*-value
IgG	-773 (-805, 689)	0.378	-146 (-533, 3457)	0.15
IgG1	-2136 (-3477, -795)	0.002	-1401 (-2571, -231)	0.019
IgG2	-1512 (-2894, -128)	0.032	-719 (-2135, 697)	0.318
IgG3	-1482(-2542, -423)	0.006	-983 (-1841, -126)	0.025
IgG4	58 (-590, 706)	0.86	195 (-436, 826)	0.542

^1 ^Univariate linear regression. ^2 ^Multiple linear regressions adjusting for the effect of age including age as square root of age. Models using other forms of age correction (age, age × age, log age) gave similar results.

### Relationship between R0-GLURP IgG and risk of developing febrile malaria in Mgome

The risk of developing a febrile malaria episode during the study decreased with age and was 65.2% (43/66), 11.5% (6/52), 11.4% (4/37) and 5.6% (1/18) for the age groups 0–4, 5–9, 10–14 and 15–19, respectively. Logistic regression models correcting for age indicated that the presence of a measurable R0-GLURP IgG1 was associated with a reduced risk of febrile malaria episodes [adjusted odds ratio (AOR) 0.39 (95% CI: 0.15, 0.99), *P *= 0.047]. The age adjusted odds ratio for individuals who had a measurable IgG3 response was 0.52, but the 95% confidence interval for this estimate was wide and not significantly different from one (Table 2). The presence of antibodies to R0-GLURP of the IgG2 or IgG4 subclasses was not associated with significant reduction in the risk of febrile malaria episodes.

**Table 2:** Odds ratio for the risk of febrile malaria episodes

R0-GLURP antibodies	Unadjusted odds ratio (95% CI)^1^	*P*-value	Adjusted odds ratio (95% CI)^2^	*P*-value
IgG	0.49 (0.25 – 0.94)	0.031	1.13 (0.50 – 2.53)	0.77
IgG1	0.17 (0.07 – 0.42)	<0.001	0.39 (0.15 – 0.99)	0.047
IgG2	0.47 (0.20 – 1.08)	0.075	1.42 (0.58 – 3.46)	0.445
IgG3	0.25 (0.11 – 0.58)	0.001	0.52 (0.20 – 1.40)	0.197
IgG4	0.42 (0.18 – 0.99)	0.046	0.77 (0.30 – 2.03)	0.602

^1 ^Logistic regression indicating risk of febrile malaria episode in individuals with detectable plasma levels of anti R0-GLURP antibodies (for IgG, IgG1, IgG2, IgG3 and IgG4, respectively) relative to those without a measurable antibody response. ^2 ^Adjusted for the effect of age including age as square root of age.

### Relationship between R0-GLURP IgG and risk of anaemia in Mgome

In logistic regression models including age and sex, there were no statistically significant associations between the risk of anaemia and having a measurable IgG [AOR: 1.03 (0.45, 2.35); *P *= 0.95] or IgG subclass (data not shown) R0-GLURP antibody response. Similarly, in linear regression models correcting for age and sex, there were no associations between plasma R0-GLURP IgG or IgG subclass levels and the haemoglobin level at enrolment (data not shown).

## Discussion

Studies in which observed clinical protection is linked to the level of malaria antibodies on an individual level have been used to identify vaccine targets [5,6,15,22]. In these types of studies, participants are often divided into susceptible and protected individuals and this obviously requires that a reasonable number of the participants develop clinical symptoms during follow up. To allow comparisons between immunological parameters, it is also preferable that the protected individuals are as closely age matched to the susceptible individuals as possible. Due to problems of obtaining sufficient amounts of plasma from infants and young children, most studies have been conducted in areas of moderate transmission targeting children between 5–15 years or in areas of high transmission targeting children over 5 years and adults. These studies have documented that the presence of R0-GLURP IgG is associated with protection against febrile malaria attacks in areas of moderate and seasonal malaria transmission [6,13-15]. The acquisition of malaria immunity is governed by the transmission intensity [23], and in areas of holoendemic transmission children of three to four years of age have already developed considerable protection against febrile episodes [14,15]. In the current study, individuals with a measurable anti R0-GLURP IgG1 response had a statistically significant reduction in the risk of getting a febrile malaria attack compared with those without such antibodies. The results cannot unravel whether GLURP antibodies directly were responsible for the effect or whether they constitute a marker for other immunological activities. The protection provided by GLURP antibodies is mainly thought to be mediated through IgG1 and IgG3 antibodies which dampen the growth of blood stage parasites by antibody dependent cellular inhibition (ADCI) [13,14,17,24]; although levels of IgG2 GLURP antibodies have also been implicated in protection [13,14]. It is, therefore, of interest that a significant association between the level of anti R0-GLURP IgG1 and IgG3 antibodies and the parasite density at enrolment has been found in this study.

The acquisition of IgG against GLURP was highly dependant on malaria transmission intensity. In the high transmission village antibody levels increased markedly after the age of two years, and in the three to four years old children a very high percentage had a detectable antibodies response. In the village with moderate transmission antibody acquisition was much slower and GLURP antibody response rates over 50% were only seen in the age groups older than 10 years.

In areas of high malaria transmission one of the major disease burdens attributable to *P. falciparum *infection is anaemia occurring in infants and young children [2,19,21]. In Mgome, all children under two years had haemoglobin levels under 11 g/dl. Thus, it was disappointing that the levels of anti R0-GLURP antibodies were not associated with haemoglobin levels in multiple linear regression models or with anaemia in multiple logistic regression models.

## Conclusion

In conclusion, this study conducted in an area of intense malaria transmission detected an association between protection against febrile malaria disease and presence of anti R0-GLURP antibodies and indicated that increasing levels of antibodies of the IgG1 and the IgG3 subclasses are associated with a reduction in *P. falciparum *parasite densities.

## Authors' contributions

JPAL carried out field surveys, performed ELISA, analysed data and drafted the manuscript. LSV carried out field surveys and in collaboration with JPAL, MA and MT contributed to the set up of the ELISA. BPM carried out field surveys and participated in data analysis. MT, AYK, MML and TGT conceived the design of the study. All authors amended and approved the final manuscript.
